# 16S rRNA phylogeny and clustering is not a reliable proxy for genome-based taxonomy in Streptomyces

**DOI:** 10.1099/mgen.0.001287

**Published:** 2024-09-10

**Authors:** Angelika B. Kiepas, Paul A. Hoskisson, Leighton Pritchard

**Affiliations:** 1 Strathclyde Institute of Pharmacy and Biomedical Sciences, University of Strathclyde, 161 Cathedral Street, Glasgow, G4 0RE, UK

**Keywords:** 16S, Actinomycetales, evolution, phylogeny, *Streptomyces*, taxonomy

## Abstract

*Streptomyces* is among the most extensively studied genera of bacteria but its complex taxonomy remains contested and is suspected to contain significant species-level misclassification. Resolving the classification of *Streptomyces* would benefit many areas of applied microbiology that rely on an accurate ground truth for grouping of related organisms, including comparative genomics-based searches for novel antimicrobials. We survey taxonomic conflicts between 16S rRNA and whole genome-based *Streptomyces* classifications using 2276 publicly available *Streptomyces* genome assemblies and 48 981 publicly available full-length 16S rRNA *Streptomyces* sequences from silva, Greengenes, Ribosomal Database Project (RDP), and NCBI (National Centre for Biotechnology Information) databases. We construct a full-length 16S gene tree for 14 239 distinct *Streptomyces* sequences that resolves three major lineages of *Streptomyces*, but whose topology is not consistent with existing taxonomic assignments. We use these sequence data to delineate 16S and whole genome landscapes for *Streptomyces*, demonstrating that 16S and whole-genome classifications are frequently in disagreement, and that 16S zero-radius Operational Taxonomic Units (zOTUs) are often inconsistent with Average Nucleotide Identity (ANI)-based taxonomy. Our results strongly imply that 16S rRNA sequence data does not map to taxonomy sufficiently well to delineate *Streptomyces* species routinely. We propose that alternative marker sequences should be adopted by the community for classification and metabarcoding. Insofar as *Streptomyces* taxonomy has been determined or supported by 16S sequence data and may in parts be in error, we also propose that reclassification of the genus by alternative approaches may benefit the *Streptomyces* community.

**Impact Statement**

*Streptomyces* spp. are a rich source of antimicrobials and biotechnologically useful enzymes. Applied microbiology methods for natural product (e.g. antibiotic) discovery, such as comparative genomics, rely on accurate taxonomic classification of related microbes to provide a ‘ground truth’ for analyses. However, *Streptomyces* taxonomy is complex and historical classifications based on the 16S rRNA gene sequence are suspected to have limitations and inaccuracies in comparison with whole-genome methods. Despite this, 16S-based classifications still frequently provide a basis for taxonomy, community composition, and strains to prioritise for study. We generate the most complete 16S phylogenetic tree for *Streptomyces* to date and investigate whole-genome Average Nucleotide Identity (ANI) and 16S sequence classifications for a diverse and comprehensive set of over 2000 *Streptomyces* genomes. We find that 16S rRNA sequences do not map well to *Streptomyces* whole-genome taxonomy. In particular, distinct *Streptomyces* species may share identical full-length 16S sequences, and related isolates belonging to the same species may share no common 16S rRNA sequence. Prevailing *Streptomyces* classifications derive in large part from 16S sequence data, and the classification presented here provides a basis for community-led reclassification of *Streptomyces* by alternative approaches.

## Data Summary

The authors confirm all supporting data, code and protocols have been provided within the article or through supplementary data files.

All code, and raw and processed supporting data for this work, is publicly available under a CC-BY 4.0 license from GitHub (https://github.com/sipbs-compbiol/Kiepas_et_al_2024_16S doi:10.5281/zenodo.11082898) and Zenodo (https://zenodo.org/records/10991761 doi:10.5281/zenodo.10991760).

The flowchart in File S28 provides an overview of analysis and serves as a guide to Supplementary Files generated during reconstruction of 16S phylogeny. The flowchart in File S29 outlines the workflow processes and supplementary materials used for analysis of 16S rRNA sequences from *Streptomyces* genomes.

## Introduction

The genus *Streptomyces* is among the most biotechnologically important groups of organisms and has been extensively screened for production of bioactive metabolites [1]. *Streptomyces* spp. are prolific producers of antimicrobial natural products that have changed the course of modern medicine through prophylaxis and treatment of infection [2,3]. However, the use of these and other antimicrobials has led inevitably to the emergence of antimicrobial resistance (AMR) in the clinic [3]. AMR-related deaths are predicted to rise to ten million annually and to become the leading cause of death worldwide by the year 2050 [4]. The efficacy of current antibiotics is under threat at the same time as discovery and development of novel medications is on the decline, so there is an urgent need to discover and develop novel antimicrobials for clinical applications [5].

Recent genome mining efforts suggest that a large number of natural products produced by the *Streptomyces* genus are yet to be identified and characterised, and it is hoped that among these novel antimicrobials may be some that could aid in combating the AMR crisis [2]. Exploring this range of potentially novel natural products has challenges. Experimental approaches have delivered most pharmaceutically useful antimicrobials [6,7], but these methods are labour intensive and often lead to rediscoveries of known antibiotics (the so-called ‘dereplication problem’) [8]. Further limitations arise from difficulties in cultivating fastidious and recalcitrant microorganisms, and inducing biosynthesis of these compounds under laboratory conditions [7,9]. In recent years mining of genome sequences for biosynthetic gene clusters (BGCs) predicted to make secondary metabolites, without the need for cultivation of microorganisms, has emerged as a powerful strategy for drug discovery [10]. Bioinformatic tools for genome mining and bioprospecting, such as antiSMASH [11], GECCO [10], ARTS [12], PRISM [13], and numerous other tools that identify BGCs by their genetic and evolutionarily conserved features have been developed [14,16]. As whole genome sequencing has become more accessible these tools have allowed the dereplication problem to be addressed rapidly at scale to prioritise strains possessing potentially novel BGCs.

Despite the increased availability of whole genome sequences, 16S rRNA remains a standard approach for taxonomic identification, especially in the context of microbial communities and metabarcoding [17]. Clustering of 16S rRNA sequences into Operational Taxonomic Units (OTUs) at specified thresholds continues to be a commonly used approach for grouping similar sequences as a proxy for taxonomic identity [18]. Thresholds proposed in 1994, when only a small fraction of the currently known 16S rRNA sequences were available, recommended that 95% sequence identity would circumscribe representatives of the same genus, and 97% sequence identity would do so at species level [18]. As bacterial genome sequencing became more common, it was realised that these thresholds were too permissive, and it was later recommended to treat each unique 16S rRNA sequence (or amplicon sequence variant, ASV) as a distinct taxonomic unit, a zero-radius Operational Taxonomic Unit (zOTU) [19,21]. However, it is known that distinct species can share identical 16S rRNA sequences, including *Streptomyces* such as *S. chrestomyceticus* and *S. paromomycinus*, and that some species contain multiple distinct copies of 16S rRNA sequences [22,23].

Comparative genomics and pangenome analysis depend on accurate preclassification of related strains, typically by common membership of a taxonomic group. Bioprospecting that uses these techniques to identify potentially novel BGCs requires accurate classification of strains of interest [24]. *Streptomyces* taxonomy is historically based on polyphasic approaches incorporating morphological characteristics, phenotypic, and single-gene phylogenetic analyses, but these analyses often struggled to provide robust phylogenies [25]. The sequence diversity of *Streptomyces* was previously demonstrated through a landmark 16S rRNA phylogeny defining more than 130 lineages within the genus, though with some poor phylogenetic resolution due to the limited length and sequence variation of the 16S gene [26]. In particular, the 16S phylogeny proposed in [27] has been found to be inconsistent with subsequent whole-genome phylogenies and distance measures [28,29]. For example, the 16S rRNA gene was found to be unable to distinguish between members of the taxonomic orders Streptomycetales and Frankiales [30,31]. To address these issues 16S was later augmented with Multi Locus Sequence Analysis (MLSA) to further resolve phylogenetic relationships within the group [32,33].

The recent availability of large-scale genome sequence data allows us to revisit phylogenetic and evolutionary relationships in complex taxa such as *Streptomyces*. Whole-genome distance measures and phylogenomic approaches now play an increasingly important role in delineating species and genera. However, there remains no consensus on the interpretation of genus or species boundaries determined by whole genome sequence similarity [34,35]. A 94–96% ANI threshold is commonly used as an operational definition of species circumscription, but interpretations of genome coverage can be difficult due to variation in genome size (affected for instance by genome expansion events, and presence or absence of plasmids) and comparisons between genomes of differing quality. Moreover, pairs of genomes with homology across less than 50% of their total genome length may even represent distinct candidate genera, regardless of ANI identity [36]. Measures such as digital DNA–DNA hybridization (dDDH), ANI, average amino acid identity (AAI) and MinHash-related approaches enable rapid estimation of taxonomic relationships based on microbial genome sequences [35,40]. Using whole genome data promises higher phylogenetic resolution than can be achieved with marker sequences, at the expense of averaging across the effects of multiple evolutionary processes [28,41]. These approaches allow detection of mislabelled genomes, but it remains important to treat whole-genome taxonomy with some caution as mislabelled and low-quality (e.g. contaminated or incomplete) genomes may give erroneous results [42].

Here we report comprehensive classification of *Streptomyces* using 16S and whole-genome distance methods, with goals to delineate the genomic landscape of this group of organisms, and to identify and interpret incongruences between the two approaches. We study all 48 981 available full-length 16S rRNA *Streptomyces* sequences and the 2276 *Streptomyces* genomes available at the time of writing. Using 14 239 distinct full-length *Streptomyces* 16S rRNA sequences, we present the most comprehensive 16S phylogeny of *Streptomyces* known to us, to date. We investigate the utility of clustering these 16S rRNA sequences into OTUs across a range of threshold identities, including previously recommended 95%, 97% and zOTU identity thresholds. Finally, we examine the distribution of individual 16S rRNA sequences across *Streptomyces* genomes to determine whether a mapping exists between 16S rRNA sequences and whole genome-derived species boundaries, with a specific goal to understand whether zOTUs are congruent with genome-based classifications.

## Methods

### Acquisition of 16S rRNA *Streptomyces* sequences from major 16S rRNA databases

The 16S rRNA sequences were manually downloaded from silva v138.1 [43], RDP v11.5 [44], NCBI [45] (New ribosomal RNA blast) PRJNA33175 and Greengenes v13.5 [46] as indicated in Table 1 (File S28).

**Table 1.** Summary description of 16S rRNA databases used in this manuscript

Database	Version	Acquisition date	No. of sequences
silva	138.1	26 October 2020	2 224 740
Greengenes	13.5	26 October 2020	1 262 982
RDP	11.5	10 November 2020	3 196 041
NCBI	PRJNA33317	10 November 2020	20 801
			**TOTAL**: 6 704 552

Of these, Greengenes is the only database that stores sequence and taxonomy information in separate files. The taxonomy file (gg_13_5_taxonomy.txt, File S2) was accessed on 26 October 2020, and taxonomic information was mapped to sequence data by matching the sequence identifier using the Python script gg_map_taxonomy.py (File S2).

### Selection of full-length *Streptomyces* 16S rRNA sequences

To obtain full-length 16S sequence candidates, we filtered all sequences from the sources above to retain only sequences having the keyword *Streptomyces* in the taxonomy field, and excluding any 16S rRNA with length less than 1200 bp. The average length of a complete 16S gene is approximately 1550 bp, and a 1200 bp lower threshold on sequence length was chosen to capture all hypervariable regions, maximising information about sequence variation, and to filter out database sequences targeting only a subset of hypervariable regions. We refer to this >1200 bp filtered set as ‘full-length’ sequences. The resulting 48 981 full-length sequences were combined to create a local 16S database (all_DNA_whole_16S_strep.fasta, File S3), and base coding was standardised to replace uracil with thymine (U->T) using the Python script get_complete_strep_seq.py (File S3).

### LPSN nomenclature validation

Standardised nomenclature data was downloaded from the List of Prokaryotic Names with Standing in Nomenclature [47] (LPSN; LPSN_taxonomy.csv, File S6) on 16 February 2023. Species-level nomenclature previously assigned to the 48 981 full-length 16S rRNA *Streptomyces* sequences in the source database(s) was validated against this list using the Python script get_NCBI_taxID_and_LPSN_status.py (File S6).

### Elimination of nomenclature disagreements at higher taxonomic ranks

In some cases, the stated taxonomy at higher ranks of a sequence was a lineage not recognised to contain *Streptomyces* spp. The nomenclature at ranks up to kingdom assigned to extracted full-length 16S rRNA *Streptomyces* sequences in the source database(s) were validated using the Python script check_nomenclature_hierarchy.py (File S3) to identify and note, but not correct, this and similar cases of nomenclature hierarchy disagreement.

### Removal of redundant, chimaeric, and ambiguous sequences

We removed (i) 24 132 strictly identical redundant sequences (--derep_fulllength) identified using vsearch v2.15.1 [48] (remove_redundancy.sh**,** File S4), (ii) eleven sequences with more than 153 ambiguity symbols, (this threshold was determined by considering the negative binomial distribution of ambiguity symbols in the dataset; for detailed information see remove_ambiguity in File S4), and (iii) 2158 chimaeric sequences identified using vsearch v2.15.1 (remove_chimeras.sh**,** File S4). These operations reduced the ≈49 000 full-length 16S rRNA *Streptomyces* database (all_DNA_whole_16S_strep.fasta) to a total of 22 680 sequences (non_chimeras.fasta, File S4).

### Clustering of complete 16S rRNA *Streptomyces* sequences

The 22 680 retained full-length *Streptomyces* sequences were clustered using vsearch 2.15.1 [48] at pairwise percentage sequence identity thresholds (--id) ranging from 98–100% in steps of 0.1% (cluster_sequences.sh, File S5). The number of distinct taxonomic assignments in a cluster for each clustering threshold was determined using the NCBI reference taxonomy [49] (downloaded 31 January 2023 from https://ftp.ncbi.nih.gov/pub/taxonomy/taxdmp.zip; names.dmp, File S6). Each sequence was assigned an NCBI taxID corresponding to the LPSN-validated taxon assigned to it in the source database(s). This process generated 14 239 zOTUs with 100% pairwise sequence identity according to the clustering threshold, although the input sequence set was non-redundant. Nomenclature and corresponding taxIDs for redundant sequences removed in the previous section were assigned to the retained representative sequence for this analysis, as multiple different taxonomic classifications were identified for many redundant sequences (cluster_composition_analysis.py, File S6).

### Phylogenetic reconstruction

Representative sequences from all 14 239 16S rRNA zOTUs, and a further ten 16S rRNA outgroup sequences belonging to isolates from *Kitasatsopora, Streptoalloteichus* and *Clavibacter* genera (outgroup.fasta, File S9) were aligned using nextalign v0.1.4 [50] against the GCF_008931305.1 16S rRNA reference sequence from *S. coelicolor* A3 (S_coelicolor_A32.fasta, File S9). Alignments were trimmed using trimAl v1.4 [51] (trim_alignments.sh, File S9), and subsequently dereplicated using RaxML (leaving 9049 sequences; alignment_dereplication.sh, File S9). A Maximum-Likelihood tree was estimated using RaxML-NG v1.0.2 [52] under the GTR_F0 model recommended by RaxML-NG with 100 Transfer Bootstrap Expectation (TBE) replicates (raxml_bootstraps.sh; raxml_tbe.sh, File S10) on the ARCHIE-WeSt High-Performance computing cluster (www.archie-west.ac.uk) based at the University of Strathclyde. Resulting trees were visualised using the ETE3 Toolkit [53].

### Assessment of unique 16S rRNA sequences from *Streptomyces* genomes

All 2276 publicly available *Streptomyces* genome sequences (streptomyces_genomes.txt, File S17) were downloaded from NCBI on 8 July 2021 (download_genomes.sh**,** File S17). We discarded 120 genomes from the analysis that were indicated as suppressed in the NCBI assembly report downloaded on 30 January 2023 from https://ftp.ncbi.nlm.nih.gov/genomes/refseq/assembly_summary_refseq_historical.txt (assembly_summary_refseq_historical.txt, check_genome_status.py, File S17). Updated versions of five genomes (streptomyces_replaced_genomes.txt, File S17) were manually downloaded from NCBI [45] on 30 January 2023. A total of 6692 16S rRNA sequences were extracted from the 2156 *Streptomyces* genomes by matching the key word ‘16S’ in the \gene_qualifiers GenBank field (extract_16S.py, File S18). We filtered the extracted 16S sequences to a total of 4227 by retaining only those sequences from the 1369 genomes (retained_genomes.txt, File S18) that exclusively contain only full-length and ambiguity symbol-free 16S sequences (filter_16S_seq.py**,** filtered_16S_seq_from_strep_genomes.fasta, File S18). All 4227 such sequences extracted from *Streptomyces* genomes were aligned using nextalign v0.1.4 [50] against the same GCF_008931305.1 16S rRNA reference sequence as used previously (S_coelicolor_A32.fasta, File S9). The alignment was trimmed using trimAl v1.4 [51] (trim_alignment.sh, File S19), and genomes sharing any identical 16S rRNA sequences were clustered (get_input_genomes_for_pyani.py, File S19) and their taxonomic boundaries determined using pyANI v0.3 [36] (pyani_analysis.sh, File S19). We adopted 50% ANI coverage and 95% ANI identity thresholds to estimate genus and species boundaries respectively.

### Network analysis of genomes based on shared 16S rRNA sequences

We constructed a network representing individual genomes as nodes and assigning edges between genomes with weights corresponding to the number of shared identical 16S sequences, to represent the 1369 *Streptomyces* genomes that contain only full-length and ambiguity symbol-free 16S rRNA sequences. Edges corresponding to pairs of genomes with no 16S sequence in common were removed such that an edge in the graph implied at least one identical 16S sequence in common. The graph was constructed and processed using NetworkX [54], and visualised interactively with plotly v5.6.0 (https://plotly.com/python/; genome_16S_NetworkX.ipynb; File S20). Node layout was calculated using Cytoscape v3.9.0 [55] (prefused force-directed layout). ANIm analysis was performed using pyANI v0.3 [34] to determine taxonomic boundaries for all genomes found in the same subgraph, as above (pyani_analysis.sh**,** File S20).

## Results and discussion

### A majority of publicly available 16S *Streptomyces* sequences are not full length, are redundant or low-quality, or have issues with taxonomic nomenclature

High-quality sequences with accurate and detailed metadata are crucial for ensuring the accuracy and reliability of research and analysis, particularly in the fields of genomics, taxonomic classification, and applied microbiology. Unintentional use of inaccurate database records can potentially lead to false interpretations and flawed research outcomes. Of the 62 482 16S rRNA sequences belonging to the genus *Streptomyces* across the silva v138.1, RDP v11.5, Greengenes v13.5 and NCBI (PRJNA33175) databases (see Methods), we determined that 48 981 (78.4%) were full-length (>1200 bp length) sequences. In total, we identified 24 849 non-redundant full-length 16S rRNA sequences across these databases: 50.7% of all full-length *Streptomyces* sequences, and 39.8% of all database *Streptomyces* sequences (File S28).

Prokaryotic taxonomic nomenclature is the primary mechanism for unambiguous communication about an organism’s identity. Correct nomenclature avoids undesirable clinical, ecological, agricultural, and pharmaceutical consequences [56,58]. The databases considered in this work rely on a variety of taxonomic authorities: RDP uses Bergey’s Manual [59,60], silva uses LPSN and Bergey’s Manual [47]; and NCBI combines nomenclature provided by the submitter with that from Greengenes, basing its nomenclature on that in the NCBI taxonomy [46,61]. All these schemes are good-faith efforts to follow the International Code of Nomenclature for Prokaryotes (ICNP) [62], but we find that records are in some cases inaccurate. Specifically, we compared the taxonomy assigned to each full-length sequence in the source database(s) against LPSN, finding that only 14 859 (30.3%) sequences had taxonomic classifications consistent with LPSN at species level. Fourteen-hundred (2.9%) sequences were annotated with synonyms of valid names, but 28 333 (57.8%) sequences were labelled only as unclassified *Streptomyces*. A further 17 sequences (0.03%) had names with typographical errors, and no record in LPSN was found at all for 4372 (8.9%) sequences. The LPSN status of all 48 981 sequences used in this manuscript is provided in complete_strep_records_info.csv (File S6).

Sequences may be annotated with synonyms of taxon names at various ranks, and it is reasonable to expect that these taxon names should be consistent within the same lineage (e.g. that a genus name is assigned to the correct family). We examined nomenclature assignments within the source databases at ranks from kingdom to genus to determine sequences with taxonomy assigned in the following ways: (i) correctly to all ranks above *Streptomyces*, eg. AWQW01000120.100.1367 belongs to Bacteria kingdom, Actinobacteriota phylum, Actinobacteria class, Streptomycetales order, Streptomycetaceae family, *Streptomyces* genus and *Streptomyces niveus* species (we note that nomenclature is fluid and, for example, at phylum level Actinobacteriota has been superseded by Actinomycetota [63], but choose to reflect the nomenclature assigned in the database); (ii) to higher ranks not expected to contain the *Streptomyces* genus (eg. KY753270.1.1450 belongs to Bacteria kingdom, Firmicutes phylum, Bacilli class, Bacillales order, Bacillaceae family, *Bacillus* genus, and *Streptomyces pseudovenezuelae* species); (iii) to higher ranks that correctly include *Streptomyces* from kingdom to genus, but where the annotated nomenclature nevertheless disagrees on the parent genus name (eg. JN987181.1.1444 belongs to Bacteria kingdom, Actinobacteriota phylum, Actinobacteria class, Streptomycetales order, Streptomycetaceae family, *Streptomyces* genus and *Lactobacillus apodemi* species); and (iv) to ambiguous hierarchies where there is a complete lack of information about higher ranks (eg. NR_042095.1). Our results identify numerous misassignments in groups (i)-(iv), consistent in general with previous observations of conflicting nomenclature at higher ranks in Greengenes and silva [64], summarised as a Sankey plot in File S30. A complete list of all 48 981 investigated sequences is given in nomenclature_hierarchy_info.csv (File S3).

Chimaeric sequences, and sequences with a high proportion of ambiguity symbols, can be destructive in phylogenetic analyses, leading to model misspecification, incorrect branch length and compromised topology estimation [65]. Eleven sequences with more than 153 nucleotide ambiguity symbols were discarded from our dataset prior to analysis. During the data cleaning process, a further 2158 potentially chimaeric sequences were also identified and removed from the dataset. Following the filtration and cleaning process, 22 680 full-length nonredundant (strictly non-identical) high-quality sequences (46% of the initial dataset) remained and were taken forward for further analyses and phylogenetic tree reconstruction. Despite significant and diligent long-term efforts by curators to remove poor quality sequences from the databases used in this manuscript [46], we still required to discard a large proportion of the original dataset to avoid introducing identifiable sources of potential inaccuracy to our analysis.

### 16S percentage sequence identity thresholds do not reliably delineate existing *Streptomyces* species assignments

The long-standing 16S rRNA clustering threshold of 97% sequence identity for species level circumscription has been robustly questioned, and current best practice is to use zOTUs, or Amplicon Sequence Variants (ASVs) for taxonomic classification and clustering of 16S and other marker genes [20]. To identify whether any 16S percentage identity clustering threshold adequately circumscribes *Streptomyces* taxa, we applied a range of threshold identities to the 22 680 full-length *Streptomyces* 16S rRNA sequences identified above to generate clusters of sequences with a minimum pairwise identity (including 14 239 zOTUs), then determined agreement between taxonomic species and cluster membership. If zOTUs are assumed to be an accurate proxy for species, then the 14 239 known zOTUs would imply the same number of candidate *Streptomyces* species. Currently at least 650 species are recognised within *Streptomyces*, so the observation of so many zOTUs might suggest either a high degree of cryptic species diversity, or that 20 or more distinct 16S rRNA sequences could be characteristic of each *Streptomyces* species.

Our initial examination of pharmaceutically important strains *S. griseus* (streptomycin producer), *S. lydicus* (natamycin, lydimycin and streptolydigin producer), *S. clavuligerus* (clavulanic acid and cephamycin C producer [1]) and the phytopathogen *Streptomyces scabiei* [66] demonstrates that there is not for every species a one-to-one mapping between taxonomic assignment and 16S rRNA zOTU. Specifically, we find that sequences annotated as *S. clavuligerus* are distributed across eight zOTUs, *S. griseus* across 145 zOTUs, *S. lydicus* across 16, and *S. scabiei* across 62. These findings are consistent with previous observations that *Streptomyces* genomes can possess multiple non-identical 16S rRNA sequences (File S26). The existence of multiple distinct 16S rRNA sequences corresponding to a single species implies that a naïve interpretation of 16S metabarcoding which assumes that zOTU diversity reflects species diversity may overestimate the number of species per sample. A naïve count of zOTUs would also be systematically biassed when quantifying the abundance of *Streptomyces* species that possess varying numbers of distinct 16S sequences.

**Fig. 1.**
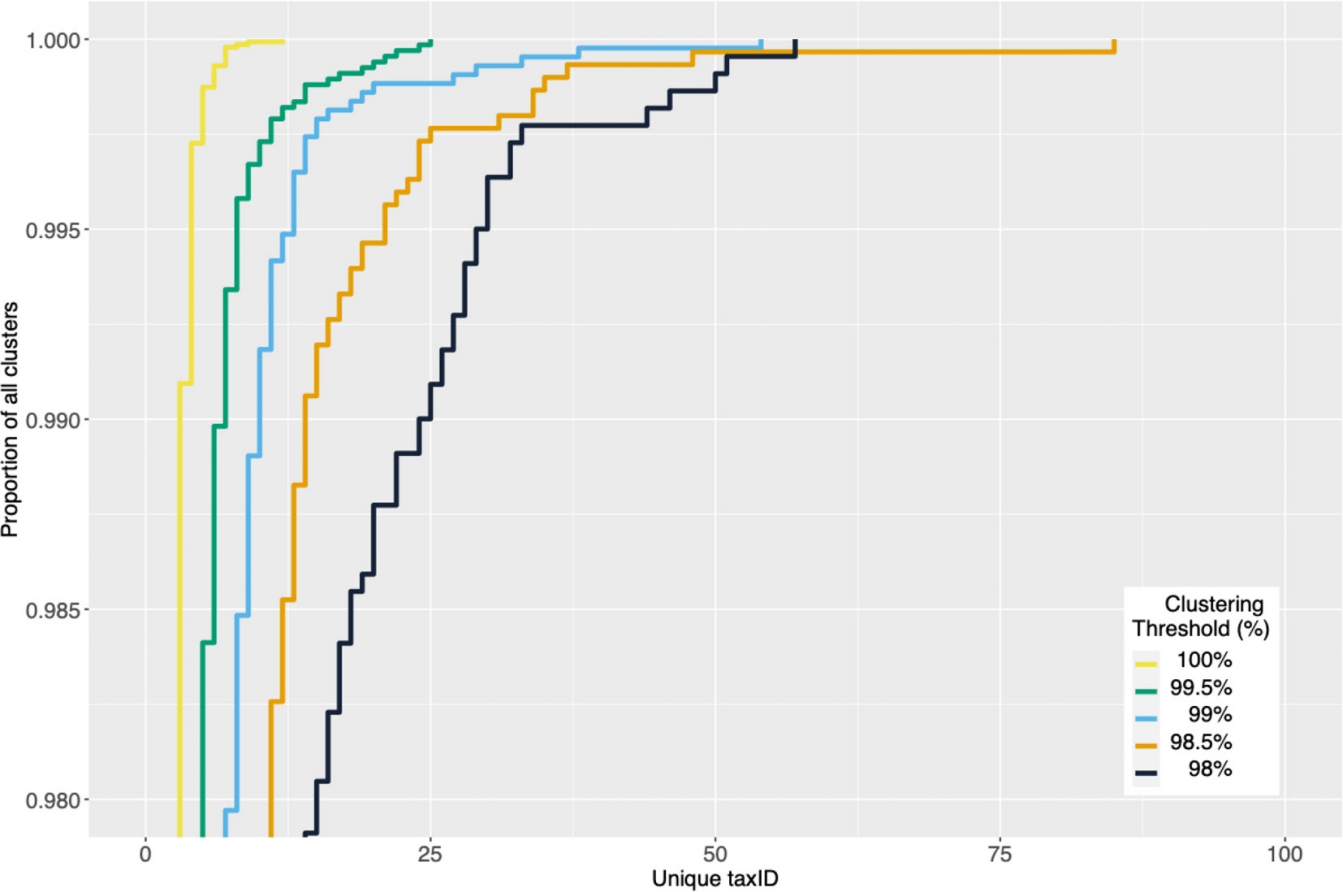
Empirical cumulative distribution of taxonomic composition (the number of unique, identifiable species-level taxIDs) at a range of pairwise percentage sequence identity clustering thresholds for full-length 16S sequences. The y-axis begins at the ninety-eighth percentile and, even for zOTUs (100% identity clustering threshold), at this level no zOTU is associated with only a single species-level taxID. At less stringent pairwise identity thresholds, the number of taxIDs observed at a given percentile is larger. The result demonstrates that a *Streptomyces* zOTU cannot in general be assumed to map to a single species-level taxonomic assignment, and that such assignments are progressively less secure as a lower sequence identity threshold is considered.

As the 16S sequence identity clustering threshold is raised systematically from 98–100% (zOTUs), we find that clusters increase in number and tend to have fewer distinct 16S sequence members (File S7). The number of assigned taxa per cluster also tends to fall as the identity threshold approaches 100% (Fig. 1). We identified 10 548 zOTUs corresponding to sequences from two or more *Streptomyces* isolates. Of these, 8326 (78.9% of all zOTUs) included at least one sequence currently named as *Streptomyces* sp. whose classification is ambiguous, and 4820 (45.7%) consisted only of such unclassified sequences. This significant proportion of unclassified sequences may correspond to distinct species, previously unclassified 16S variants in known isolates, or even unclassified members of known species, including any other species represented in the same zOTU. These sequences introduce ambiguity and disrupt estimation of the true taxonomic accuracy of 16S marker sequences.

Nevertheless, if 16S rRNA sequences do provide sufficient sequence diversity to distinguish between *Streptomyces* species, and zOTUs are a reliable proxy for taxonomic assignment at or below species-level in *Streptomyces,* we should then expect each zOTU cluster to contain only sequences annotated with the same, single, species (or to be yet unassigned *Streptomyces* sp.). However, our analysis shows that 3747 (26%) of the zOTUs we find contain 16S sequences currently assigned to at least two distinct *Streptomyces* species (File S8; cluster_taxID_info.csv, File S6). One zOTU is notably associated with up to twelve distinct species (*S. coelicolor, S. albidoflavus, S. somaliensis, S. rutgersensis, S. paulus, S. limosus, S. griseochromogenes, S. sampsonii, S. resistommycificus, S. felleus, S. violascens* and *S*. sp.). We therefore find that a substantial fraction of full-length 16S zOTUs do not map exactly to a single *Streptomyces* species assignment, and we find many examples where single 16S sequences map to multiple distinct species.

### A comprehensive *Streptomyces* 16S phylogeny

**Fig. 2.**
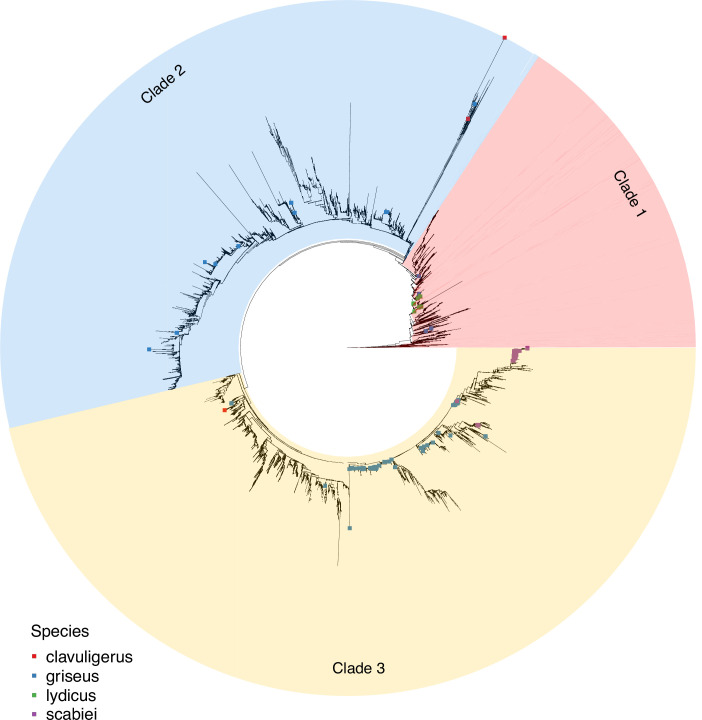
Maximum-likelihood tree of the genus *Streptomyces* constructed from 9049 full-length 16S rRNA sequences. Clades containing a single assigned species-level taxon were collapsed to single leaf nodes. Three major clades (Clade 1–Clade 3) are highlighted in distinct colours. Squares indicate 16S sequences assigned to the same species names in the source database(s): *S. griseus* sequences in blue, *S. clavuligerus* in red, *S. lydicus* in green, and *S. scabei* in purple. Sequences with these species assignments tend not to be monophyletic, indicating incongruence between taxonomy and the 16S gene tree. An equivalent rectangular phylogram is provided in File S31.

To estimate evolutionary relationships among *Streptomyces* all 14 239 zOTU sequences were used to produce a multiple sequence alignment (MSA) against a reference sequence from *S. coelicolor* A3(2). Ten related outgroup 16S sequences extracted from related non-*Streptomyces* genera were added to aid in root placement (File S9). The MSA was trimmed to a length of 1086 nucleotides and 9049 sequences after redundant sequences and sites were removed (no positions in the alignment were absolutely conserved), and a maximum-likelihood tree was calculated. Clades containing a single species taxonomic assignment were collapsed to single leaf nodes to produce a tree with 5064 nodes and facilitate visualisation (Fig. 2; full tree provided in newick format in File S10; 04_TBE.raxml.support; collapsed newick file version provided in File S11; collapsed_strep_tbe.new).

To our knowledge this is the largest, most comprehensive 16S rRNA phylogenetic reconstruction attempted for *Streptomyces* to date. No clade received TBE support greater than 60% and only 18 clades have TBE support above 50%, hence we do not consider the topology of this tree to be robust as presented. File S12 describes a phylogenetic tree where clades having TBE value of 50% or higher are marked.

A notable feature of the tree is the division of *Streptomyces* into three major clades (Fig. 2). If 16S sequence database sequences were simply consistent with the true *Streptomyces* species tree, we should expect to find that 16S sequences taxonomically assigned to the same species are broadly monophyletic (while allowing for ancestral duplications and subsequent variation, given that a large proportion of *Streptomyces* species contain multiple distinct 16S loci). Departure from this pattern might reflect unreliability in the existing taxonomic classification, or unsuitability of 16S for phylogenetic reconstruction and taxonomic placement. To examine this, we mapped pharmaceutically and agriculturally important isolates to the comprehensive 16S ml phylogeny in Fig. 2. We find that for some species 16S sequences are consistently monophyletic (e.g. *S. lydicus, S. albus* and *S. venezuelae*, File S14)*,* while some exhibit scattering within a major clade consistent with limited misannotation (e.g. *S. scabiei*, *S. lavendulae* and *S. rimosus*: File S16). However, we find some species-level taxa to be distributed widely across the tree, and even present in two or more major clades (e.g. *S. griseus, S. albus*: File S13). Many other species are represented by an insufficient number of sequences in the tree to be certain of either monophyly or widespread scattering (e.g. *S. clavuligerus* or *S. coelicolor*: File S15).

Recent reclassification of the *Streptomycetaceae* family led to the proposal of six novel genera [33]. To determine whether the proposed novel genera are resolved in our analysis, we checked their distribution in the 16S ML phylogeny. We find that members of *Wenjulia* (File S36) are placed together on the comprehensive 16S ML phylogeny, whereas sequences that would be annotated as members of *Actinacidiphila* are generally grouped together, but with some representatives distributed across the tree (File S33) Our analysis did not include any sequences that would be annotated in the *Peterkaempfera* genus, and there were too few available sequences for *Mangrovactinospora* (File S35), *Phaeacidiphilus* (File S34) and *Streptantibioticus* (File S37) to establish whether they were monophyletic or scattered.

Overall, we find evidence of taxonomic misassignment across the full scope of 16S sequences, consistent with estimations of a sequence misannotation rate for silva and GreenGenes of around 17% at ranks up to phylum, and a similar misannotation rate of 10% in RDP [64]. It is likely that, in at least some cases, apparent dispersion of a single taxon across the tree could be the result of limited sequence variation within the 16S rRNA and failure to obtain a robust phylogeny (most internal nodes have a TBE support value of lower than 50%, implying that multiple topologies are plausible, given the sequence data; Fig. 2). However, the three major clades do appear to be robustly distinguished in our phylogeny, and where the same taxon is represented in two or more of these clades we consider that this calls into question the taxonomic assignment.

### Whole-genome sequence classification indicates that distinct *Streptomyces* species can share identical full-length 16S sequences

We were unable to recover reliably strain information linking sequenced genomes to the 16S rRNA sequences from silva, Greengenes, RDP or NCBI new ribosomal RNA blast databases. Therefore, to map 16S sequence variation accurately against whole-genome classification for *Streptomyces*, we extracted 6692 16S rRNA full-length sequences from 2156 publicly available *Streptomyces* genomes. Eighty-seven of these assemblies lacked identifiable 16S rRNA sequences, and 700 genomes contained at least one 16S rRNA sequence with ambiguity symbols or partial sequences that could lead to biassed observations and overestimation of the intragenomic diversity of 16S rRNA sequences. These genomes were therefore excluded from our analysis, yielding a dataset comprising 4227 full-length, ambiguity symbol-free 16S rRNA sequences from 1369 genomes.

*Streptomyces* genomes most commonly contain six copies of 16S rRNA operons [67]. Across the 1369 assemblies analysed we find that 16S rRNA sequence copy number varies between one and twelve copies per genome (File S26). We found that 359 (26.2%) assemblies contained six copies of 16S rRNA, 144 (10.5%) had more than six copies, and 865 (63.1 %) fewer than six. Three hundred and seventy-five (27.4%) of the assemblies contained multiple non-identical 16S rRNA sequences. A single assembly (GCF_900199205.1) was found to contain eight distinct 16S rRNA sequences, but most genomes (993, 72.5%) possessed only a single 16S rRNA sequence variant, and 811 of these contained only one detectable copy of 16S rRNA.

Inconsistency in the number of 16S rRNA operons and their intragenomic heterogeneity has several possible explanations, but in many cases the variation is potentially due to *Streptomyces* genomes being assembled to different levels of completeness and quality (eg. contig, scaffold, complete or chromosome). Assemblies might also be affected by the presence of duplication artefacts or collapsed 16S rRNA sequences resulting from sequencing errors such as overassembly of short reads from distinct 16S rRNA loci into a single artefactual 16S sequence. We might then assume that NCBI complete and chromosomal *Streptomyces* assemblies more closely reflect the true genomic heterogeneity of 16S rRNA sequences. If this were the case, it would imply that 69% of *Streptomyces* isolates are likely to contain multiple distinct 16S rRNA sequences (File S27). This proportion is consistent with previous observations that *Streptomyces* strains may contain multiple distinct 16S copies, but would be twice the rate observed across all *Streptomyces* genome assemblies in NCBI [66].

To further investigate the potential scale of inaccurate *Streptomyces* taxonomic assignment when using 16S rRNA sequences, we examined the relationship between distinct genomic 16S sequences and the species assignments of their corresponding genomes. We examined the distribution of distinct 16S sequences by the number of genomes they occur in, and the number of uniquely assigned species names in NCBI associated with those genomes (File S32). We find that a single 16S sequence variant may be represented in as many as 33 genomes, and be associated with a set of genomes that are, collectively, assigned as many as six different species names.

Previous whole-genome analyses of *Streptomyces* observed that identical 16S rRNA sequences are present in strains assigned to different species [68]. Some of the discrepancy may arise from differing approaches to taxonomic assignment over the period of sequencing that might lead to, for example, the same strain being assigned to a different species depending on when the analysis was done. However, at least some of these observations may genuinely reflect that a common 16S sequence is shared across species boundaries.

For *Streptomyces* species multiple distinct 16S rRNA sequences can be found within the same genome (File S26), implying that there is a one-to-many mapping between *Streptomyces* species and 16S rRNA sequence. It follows that it is not always possible to cluster *Streptomyces* 16S sequence data without splitting a single organism into multiple zOTUs. As noted earlier, this implies that simple counts of *Streptomyces* zOTUs when metabarcoding with 16S may thus overestimate species counts and abundances. It also follows that comprehensive 16S rRNA gene trees reflect gene histories and may not recapitulate accurate species trees.

**Fig. 3.**
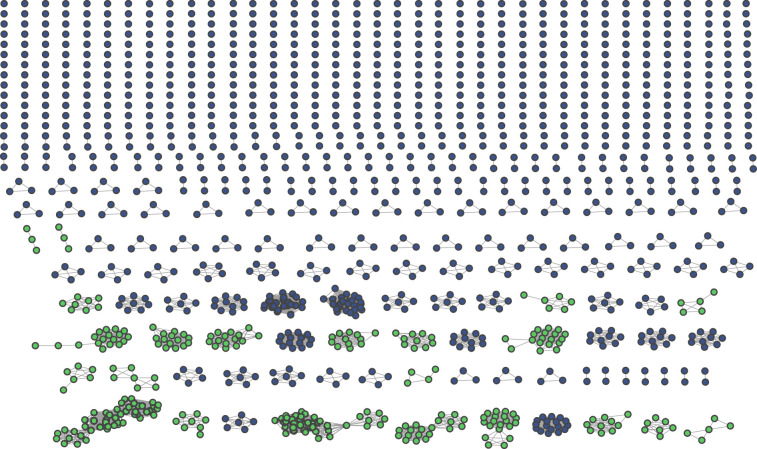
Network graph of 1369 *Streptomyces* genomes, linked by common 16S sequence. There are 709 discrete subgraphs. Each node represents a distinct genome assembly. Each edge corresponds to at least one identical 16S sequence being shared between that pair of genomes. Blue connected components form cliques in which every genome shares at least one identical 16S sequence with all other genomes in the same connected component. Green connected components do not have this property.

To investigate this relationship further, we constructed network graphs from genome-derived 16S sequences to visually represent connections between *Streptomyces* genomes based on their common 16S sequences, and thereby interpret the relationship of this network to whole-genome similarity-based species classifications. Each of the 1369 Streptomyces genomes identified above is represented as a node in each graph, and two genomes are connected by an edge in each graph if they share at least one identical full-length 16S rRNA sequence. Our network analysis resolves the 1369 *Streptomyces* genomes into 709 connected components (Fig. 3). The largest connected component unites 47 genomes, but 527 (74.3%) genomes are singletons, indicating that they share no 16S sequence with any other available *Streptomyces* assembly (an interactive version of this graph is provided in File S22).

If a direct 1 : 1 mapping existed between 16S rRNA sequences and species, then we should expect connected components to form cliques (*k*-complete graphs) where each genome within a single connected component would be linked to every other genome in the same component by at least one edge and all members of the clique would belong to the same species. However, 22 connected components formed non-cliques (Fig. 3), so some genomes within a single connected component do not share any identical 16S sequence with some of the other genomes in the same component. If all members of a connected component truly belong to the same taxon, this would imply that two members of the same taxon might share no 16S sequence in common. Alternatively, some genomes from distinct taxa may share at least one identical 16S rRNA sequence, perhaps resulting in multiple species being found within a single connected group of genomes. Such a situation might arise for any of several reasons, including inter-species recombination [69,70] or selective pressures (e.g. antimicrobials produced by competitors) that act upon the ribosome [71].

**Fig. 4.**
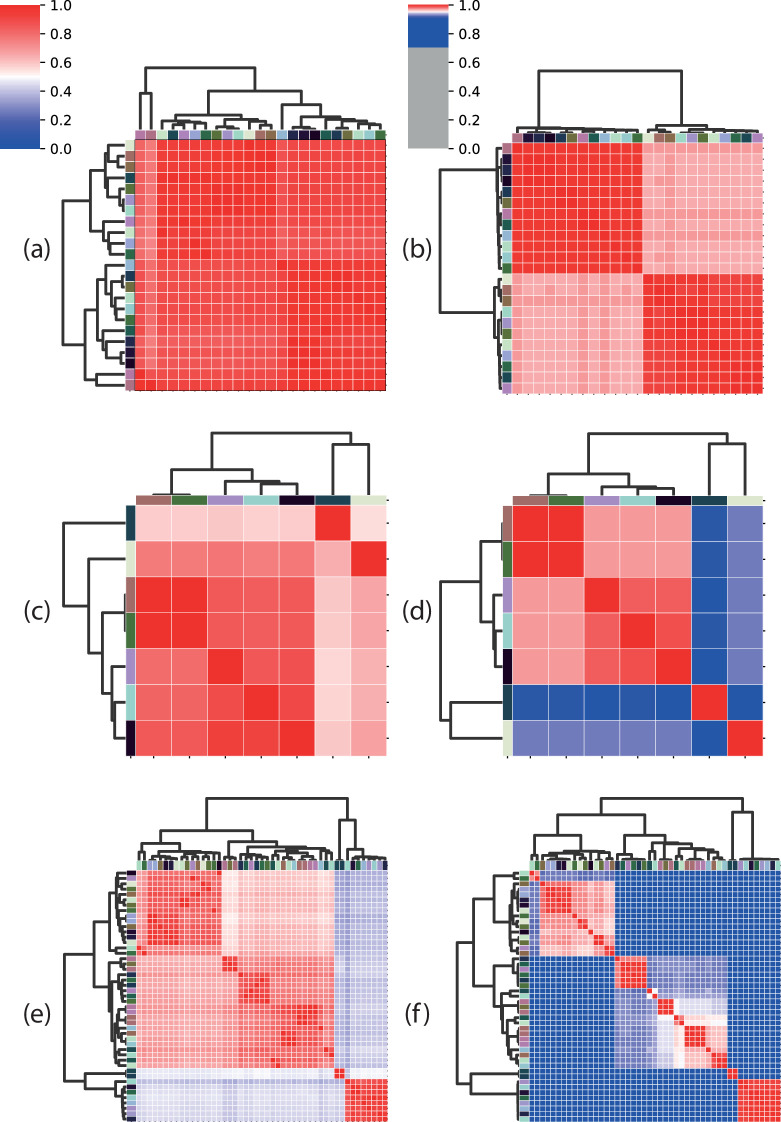
Heatmaps of ANIm coverage (left), and ANIm identity (right) for three example connected components from Fig. 3. Heatmaps in the same row correspond to comparisons for the same connected component. The left column represents percentage genome coverage, the right column %ANI. Red cells in coverage plots correspond to genome coverage of 50% or above, interpreted as common membership of the same genus; blue cells correspond to coverage below 50% and imply distinct genus assignments. In ANI plots, red cells correspond to genome identity of 95% or above, interpreted as membership of the same species; blue cells represent imply distinct species. Here, connected components correspond to: (**a, b**) genomes from a single genus and species (solid red heatmap for both coverage and identity), (**c, d**) distinct species belonging to the same genus (solid red heatmap for coverage, some pairwise identities below 95%), or (**e, f**) distinct genera and species (some pairwise coverage and some pairwise identities below 50%). ANIm coverage and identity plots for the remaining connected components are provided in Supplementary File 20, and the numbers of subgraphs falling into each category is summarized in Table S1.

We performed ANI analysis on the genomes comprising each separate connected component to establish whether the subgraph corresponded to a single grouping of genomes at genus or species level as determined by whole-genome comparison. We defined genomes as belonging to the same candidate genus if they shared at least 50% genome coverage, and belonging to the same species if they shared at least 95% ANI. These boundaries are approximations but correspond to commonly used heuristics [35]. We did not find that connected components always represent only a single kind of taxonomic relationship between its members (Fig. 4).

**Fig. 5.**
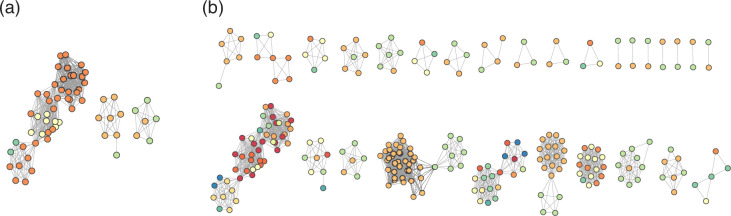
Connected components describing assemblies from distinct candidate genera (**a**) and species (**b**) Nodes represent distinct genome assemblies, while edges indicate the presence of at least one identical 16S sequence shared between the corresponding pair of genomes. Each unique candidate genus (**a**) or species (**b**) is represented as a distinct node colour.

We find that 179 (98.35%) non-singleton subgraphs correspond to genomes likely belonging to the same genus (File S23). However, three connected components (Fig. 5a) appear to comprise assemblies from distinct candidate genera. Using our whole-genome comparison threshold to define genus, and notwithstanding that the prevalence of such groups is low, we find that full-length 16S sequences are strictly not always sufficient to resolve *Streptomyces* at genus level. We find, with a similar analysis using %ANIm identity (File S24), that the majority (84%) of connected components likely represent a single species. However, 28 connected components (Fig. 5b) contain assemblies representing multiple species and we conclude that, strictly, 16S sequences cannot be guaranteed to resolve to *Streptomyces* species.

Our data are consistent with previous observations that distinct *Streptomyces* species confirmed by ANI may share identical 16S rRNA sequences [28], but our results extend these observations to demonstrate that some networks of genomes which would be assigned as the same species using ANI do not form cliques linked by identical 16S sequences. This implies that, in some cases, genomes assigned to the same *Streptomyces* species by whole-genome methods do not share *any* identical 16S sequences. Thus, in *Streptomyces*, there is a one-to-many mapping from species to 16S sequence, and a one-to-many mapping from 16S sequence to species. Taken together, our results demonstrate that use of 16S rRNA sequences in isolation for taxonomic classification of *Streptomyces* (as is often the case in 16S metabarcoding) can lead to a significant minority of misclassifications not just at the species level, as might be expected, but also at genus level.

**Fig. 6.**
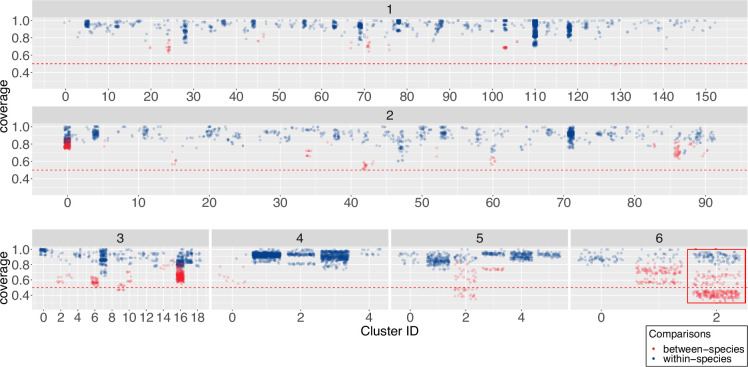
Scatterplots showing genome coverage for pairwise ANI comparisons of genomes sharing identical full-length and ambiguity symbol-free 16S sequences. The number of unique NCBI taxonomy-derived species names per cluster is displayed at the top of each subgrouping, and the red horizontal line at 50% indicates the whole-genome genus circumscription threshold. Within-species pairwise comparisons (>≈95% genome identity) are shown in blue, and between-species comparisons (<95% genome identity) are shown in red. The cluster uniting genomes with the lowest genome coverage is outlined in the red box.

**Fig. 7.**
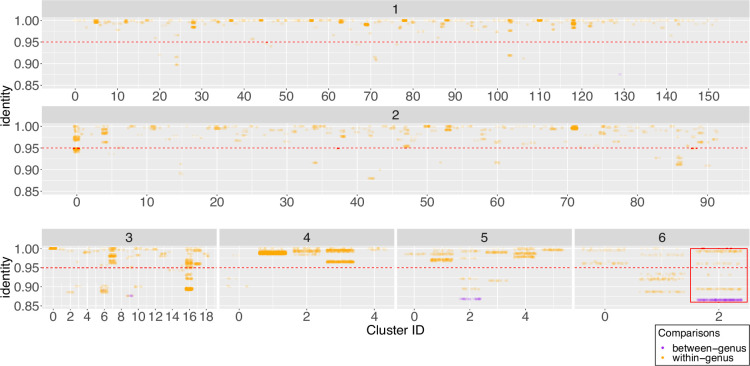
Scatterplots showing genome identity for pairwise ANI comparisons of genomes sharing identical full-length and ambiguity symbol free 16S sequences. The number of unique NCBI taxonomy-derived species names per cluster is displayed at the top of each plot, and the red horizontal line at 95% indicates the whole-genome species circumscription threshold. Within-genus comparisons (>≈50% genome coverage) are shown in orange, and between-genus comparisons (<50% genome coverage) are shown in purple. The cluster uniting genomes with the lowest genome identity is outlined in the red box.

To further delineate the relationship between 16S sequence variation and whole genome taxonomy, we measured relatedness within each connected component subgraph using ANIm (File S32). As before, we classified pairs of genomes as belonging to the same genus if they share at least 50% ANIm coverage, and belonging to the same species if they share at least 95% ANIm identity, as before. We show the pairwise comparison results as 1D scatter plots of pairwise genome coverage (Fig. 6) and pairwise genome identity for each subgraph (Fig. 7). In each figure we subdivide the subgraphs into groups corresponding to the number of distinct species currently annotated and assigned to the *Streptomyces* genomes in that subgraph (we find clusters that contain up to six distinct assigned *Streptomyces* species). We further overlay whole-genome comparison information by colouring pairwise genome comparisons according to whether they correspond to distinct genera or species by our ANIm thresholds.

We find four (1.4%) clusters containing assemblies that share less than 50% genome coverage, some with as little as 32% coverage (Cluster 2, assigned six unique taxon names; Fig. 6 – red box), and that it is rare, but possible, for genomes from different candidate *Streptomyces* genera to share at least one identical full-length 16S rRNA sequence. We also find that 36 (13%) clusters include assemblies sharing less than 95% ANI (as low as 86% ANI in Cluster two with six unique taxon names; Fig. 7 – red box). This observation is especially evident in, but not restricted to, clusters whose genomes have already been assigned distinct species names in NCBI. These results again show a one-to-many mapping between 16S sequence and *Streptomyces* genera and species as determined by whole-genome comparison, and that a unique assignment of taxonomy based only on 16S rRNA sequences cannot be guaranteed.

However frequent this potential for misclassification, our data indicate that 98.6% of clusters comprise only representatives from a single genus, and 86% representatives of a single species, as determined by whole-genome comparison (though this may not be shown in the current taxonomic assignment). Our *Streptomyces* genome sample is large but not exhaustive so this may truly reflect that 16S rRNA sequences are most often unique to a single species or genus, but it remains possible that some of these 16S sequences may also be found in as yet unsequenced genomes with a different taxonomic classification. We note that some *Streptomyces* appear to be classified with a high degree of precision, possibly due to their industrial or medical importance: members of cluster 139, representing 27 genomes currently assigned to *Streptomyces clavuligerus*, share 96% coverage, and 100% identity. *S. clavuligerus* is an industrially important organism due to its ability to produce clavulanic acid [72]. By contrast, individual members of 125 (45%) clusters seem to have been taxonomically assigned incorrectly to distinct species, as all members of the cluster share at least 50% genome coverage and 95% identity. Overall, our data show that, while many 16S rRNA sequences do resolve to single species level, there is not a precise one-to-one mapping between 16S and whole-genome taxonomy and, in general, 16S does not provide sufficient resolution to guarantee discrimination between *Streptomyces* species. We also conclude, on the basis of our observations, that extensive reclassification of published data, and potentially also revision of the genus *Streptomyces*, is required. This result is in line with recent work based on a smaller dataset of 456 strains, suggesting that there are at least six validly describable genera currently assigned as *Streptomyces* [33].

## Conclusions and recommendations

The 16S rRNA gene is one of the most widely used phylogenetic markers for studying microbial diversity due to its substitution rate and ubiquitous distribution across all bacterial species [73,74]. The 16S rRNA sequences are often clustered into OTUs at a canonical threshold of 97%, or as zOTUs/ASVs, as proxies for species [20]. With large genomic datasets now available for *Streptomyces* species, we set out to address three questions: are 16S database sequences and taxonomic annotations of sufficient quality for use as a reference in taxonomic classification; is 16S taxonomic classification a reasonable proxy for whole-genome classification in principle; and what does the whole-genome classification tell us about *Streptomyces* taxonomy in general?

We collated *Streptomyces* 16S data from Greengenes, RDP, silva and NCBI databases, reducing a total dataset of ≈48 000 sequences to a high-quality dataset of ≈14 000 non-redundant full-length sequences, excluding around two-thirds of the public sequence data on the grounds of sequence or annotation quality. The lack of available metadata for 16S sequences was a further impediment to making best use of the data that was collected, and prevented association of this data with sequenced genomes. We used Maximum Likelihood phylogeny of this high-quality sequence set to obtain the most comprehensive 16S sequence tree published to date. Despite a relatively low level of bootstrap confidence, we found a clear partitioning of *Streptomyces* into three major clades. In some cases sequence database taxonomic assignments placed the same species names at points in the topology inconsistent with a common lineage, even placing representatives of the same species into more than one major clade. By clustering zOTUs, we observed that unique 16S sequences are often associated with more than one current database taxonomic assignment, and that unique 16S sequences can often be associated with more than one *Streptomyces* species, or even candidate genus, when whole-genome approaches are used for classification. By surveying complete *Streptomyces* genomes, we identified the distribution of 16S rRNA sequences in sequenced isolates, finding numerous cases where a single genome contains several distinct 16S sequences. Taken together, these results demonstrate that there is a one-to-many relationship between 16S rRNA sequence and *Streptomyces* species, and a one-to-many relationship between *Streptomyces* species and 16S rRNA sequence. This calls into question the accuracy of 16S marker sequences for colony identification, metabarcoding, and environmental analyses in *Streptomyces*.

Finally, our whole-genome analyses of *Streptomyces* genomes demonstrated that there is an implicit underestimation of genome-level diversity in the *Streptomyces* taxonomy. We found multiple groups of genomes, currently united in the *Streptomyces* genus, that are dissimilar at the same level as genomes which are accepted to be distinct genera in other lineages of bacteria. We additionally find that, using a heuristic threshold for ANIm-based species assignment, there are grounds for reassignment of many genomes, including the unification and creation of new species-level and genus-level groupings. Our data thus suggest that revision of the *Streptomyces*, using whole-genome methods, is essential for accurate comparative genomics in this genus.

The 16S and whole-genome methods are, of course, not the only available approaches to taxonomic classification for bacteria. Multilocus sequence typing (MLST) and multilocus sequence analysis (MLSA) each employ more sequence information than the use of 16S as a single marker gene, but less than is available through whole-genome sequencing. These techniques are potentially more precise than 16S-based taxonomic assignment. Studies in *Streptomyces* using these approaches find that while MLSA analysis enhances phylogenetic resolution for some species, like *S. griseus*, it does not completely resolve conflicts within the genus [75,76]. Additionally, the discriminatory power of MLST/MLSA methods across the full range of *Streptomyces* has not been systematically surveyed, and further in-depth analysis is needed to determine definitively whether they are consistent with whole-genome taxonomy. Core genome MLSA (cgMLSA) has proved useful in other lineages, distinguishing robustly between distinct taxa in taxonomically complex genera like *Vibrio* and *Lactobacillus* [77,78]. It is likely that studying *Streptomyces* diversity using MLST/MLSA and core/whole genome methods may help resolve taxonomic conflicts within the *Streptomyces* genus, improving our assessment of diversity and the ability of comparative genomics methods to identify novel products and drugs in organisms that would otherwise be overlooked.

### Summary – to rename or not?

We have noted that several *Streptomyces* isolates and groups of isolates could, if classified in the same way as for other groups of bacteria, be reassigned to different or novel species, and even distinct genera. Increasing the number of available high-quality *Streptomyces* genome sequences would further give confidence to any such attempts at reclassification and improve our view onto the taxonomic landscape of this group, and support more robust genomic research. However, it is important to retain continuity across the literature, and to take into account practical consequences of nomenclature changes, in the light of careful community judgement [79].

Reclassification of organisms on the basis of genomic data alone has led to controversial decisions with potential for real harm. For example, renaming *Ochrobactrum* spp. (occasional opportunistic pathogens) to *Brucella* spp. (highly infectious, notifiable pathogens) has raised concerns due to the significant difference in aetiology, diagnosis, treatment and prophylaxis for the two organisms [58]. A similarly contentious situation has also arisen in the *Mycobacterium* genus [80]. Caution regarding nomenclature changes is certainly warranted.

There is perhaps too wide a range of taxonomic opinions across *Streptomyces* and other complex bacterial lineages to hope for complete agreement within the field. But there is cause for optimism. The modern age of genome sequencing brings with it ever more precise taxonomic assignment and frequent renaming of strains and taxa, breaking continuity with historical literature and community practice. Yet it also provides a potential solution to these difficulties through whole-genome strain classification approaches that are independent of taxonomy and nomenclature, such as LINgroups and genomeRxiv. These approaches are able to resolve alternative taxonomic classifications in the context of a neutral genome distance-based framework [81,82]. Classification of prokaryotes is always at some level a value judgement and, even with perfect sequence data and extensive phenotyping and metadata, we might not ever be able to agree entirely on our taxonomic language. But maybe in future we will be better able to translate between different opinions.

## Abbreviations

AAI: Average Amino Acid Identity
AMR: Antimicrobial Resistance
ANI: Average Nucleotide Identity
ASV: Amplicon Sequence Variant
BGC: Biosynthetic Gene Cluster
cgMLSA: Core Genome Multilocus Sequence Analysis
dDDH: Digital DNA-DNA Hybridisation
ICNP: International Code of Nomenclature for Prokaryotes
LPSN: List of Prokaryotic Names with Standing in Nomenclature
ML: Maximum-Likelihood
MLSA: Multilocus Sequence Analysis
MLST: Multilocus Sequence Typing
MSA: Multiple Sequence Alignment
NCBI: National Center for Biotechnology Information
OTU: Operational Taxonomic Unit
RDP: Ribosomal Database Project
16S rRNA: 16S ribosomal RNA
TBE: Transfer Bootstrap Expectation
zOTU: Zero-radius Operational Taxonomic Unit
